# Clinical antiviral efficacy of favipiravir in early COVID-19 (PLATCOV): an open-label, randomised, controlled, adaptive platform trial

**DOI:** 10.1186/s12879-023-08835-3

**Published:** 2024-01-15

**Authors:** Viravarn Luvira, William H. K. Schilling, Podjanee Jittamala, James A. Watson, Simon Boyd, Tanaya Siripoon, Thundon Ngamprasertchai, Pedro J. Almeida, Maneerat Ekkapongpisit, Cintia Cruz, James J. Callery, Shivani Singh, Runch Tuntipaiboontana, Varaporn Kruabkontho, Thatsanun Ngernseng, Jaruwan Tubprasert, Mohammad Yazid Abdad, Srisuda Keayarsa, Wanassanan Madmanee, Renato S. Aguiar, Franciele M. Santos, Pongtorn Hanboonkunupakarn, Borimas Hanboonkunupakarn, Kittiyod Poovorawan, Mallika Imwong, Walter R. J. Taylor, Vasin Chotivanich, Kesinee Chotivanich, Sasithon Pukrittayakamee, Arjen M. Dondorp, Nicholas P. J. Day, Mauro M. Teixeira, Watcharapong Piyaphanee, Weerapong Phumratanaprapin, Nicholas J. White, the PLATCOV Collaborative Group

**Affiliations:** 1 Department of Clinical Tropical Medicine, Faculty of Tropical Medicine, Mahidol University, Bangkok, Thailand; 2 Mahidol Oxford Tropical Medicine Research Unit, Faculty of Tropical Medicine, Mahidol University, Bangkok, Thailand; 3 Centre for Tropical Medicine and Global Health, Nuffield Department of Medicine, University of Oxford, Oxford, UK; 4 Department of Tropical Hygiene, Faculty of Tropical Medicine, Mahidol University, Bangkok, Thailand; 5 Clinical Research Unit, Center for Advanced and Innovative Therapies, Universidade Federal de Minas Gerais, Belo Horizonte, Brazil; 6 Department of Genetics, Ecology and Evolution, Institute of Biological Sciences, Universidade Federal de Minas Gerais, Belo Horizonte, Brazil; 7 Bangplee Hospital, Ministry of Public Health, Bangplee, Thailand; 8 Department of Molecular Tropical Medicine and Genetics, Faculty of Tropical Medicine, Mahidol University, Bangkok, Thailand; 9 Faculty of Medicine, Navamindradhiraj University, Bangkok, Thailand

**Keywords:** Favipiravir, SARS-CoV-2, COVID-19, Early treatment, Antiviral efficacy, Pharmacometrics

## Abstract

**Brief summary:**

In early symptomatic COVID-19 treatment, high dose oral favipiravir did not accelerate viral clearance.

**Background:**

Favipiravir, an anti-influenza drug, has in vitro antiviral activity against SARS-CoV-2. Clinical trial evidence to date is inconclusive. Favipiravir has been recommended for the treatment of COVID-19 in some countries.

**Methods:**

In a multicentre open-label, randomised, controlled, adaptive platform trial, low-risk adult patients with early symptomatic COVID-19 were randomised to one of ten treatment arms including high dose oral favipiravir (3.6g on day 0 followed by 1.6g daily to complete 7 days treatment) or no study drug. The primary outcome was the rate of viral clearance (derived under a linear mixed-effects model from the daily log_10_ viral densities in standardised duplicate oropharyngeal swab eluates taken daily over 8 days [18 swabs per patient]), assessed in a modified intention-to-treat population (mITT). The safety population included all patients who received at least one dose of the allocated intervention. This ongoing adaptive platform trial was registered at ClinicalTrials.gov (NCT05041907) on 13/09/2021.

**Results:**

In the final analysis, the mITT population contained data from 114 patients randomised to favipiravir and 126 patients randomised concurrently to no study drug. Under the linear mixed-effects model fitted to all oropharyngeal viral density estimates in the first 8 days from randomisation (4,318 swabs), there was no difference in the rate of viral clearance between patients given favipiravir and patients receiving no study drug; a -1% (95% credible interval: -14 to 14%) difference. High dose favipiravir was well-tolerated.

**Interpretation:**

Favipiravir does not accelerate viral clearance in early symptomatic COVID-19. The viral clearance rate estimated from quantitative measurements of oropharyngeal eluate viral densities assesses the antiviral efficacy of drugs in vivo with comparatively few studied patients.

**Supplementary Information:**

The online version contains supplementary material available at 10.1186/s12879-023-08835-3.

## Research in context

**Evidence before this study**

• The in vivo antiviral effect of favipiravir in patients with early symptomatic COVID-19 was not known.

**Added value of this study**

• High-dose favipiravir did not demonstrate antiviral activity in early symptomatic COVID-19.

• The rate of viral clearance derived from frequent oropharyngeal swabbing in early COVID-19 can be used to characterise in vivo antiviral efficacy.

**Implications of all available evidence**

• In vivo antiviral activity of COVID-19 therapeutics should be used to inform policies and practices.

**Supplementary Information:**

The online version contains supplementary material available at 10.1186/s12879-023-08835-3.

## Introduction

Favipiravir was developed in 2002 as an anti-influenza medication [1]. It is a pyrazinecarboxamide derivative, a prodrug that is metabolised within cells to its active antiviral form, favipiravir-ribofuranosyl-5'-triphosphate (favipiravir-RTP). Favipiravir-RTP is a nucleoside analogue which selectively inhibits viral RNA-dependent RNA polymerase and has shown in vitro activity against many RNA viruses [2]. Favipiravir has been licensed in Japan for influenza, and in China for investigational use, but it has not been licensed elsewhere. Favipiravir has been used in influenza at two doses- an initial dose of 3.2g (D0) followed by 1.2g daily thereafter, and a higher dose of 3.6g D0 and 1.6g daily (which is the dose used in this study). A trial using much higher doses of favipiravir (6g D0, and 2.4g daily D1-9) was conducted in patients with Ebola virus disease in Guinea, although the study had no control arm and could not reach conclusions on efficacy [3].

Favipiravir was identified as having antiviral activity against the SARS-CoV-2 virus through early in vitro screening [4–6], albeit at concentrations up to 1,000 fold higher than those required to inhibit influenza in vitro [7]. Studies in hamsters have demonstrated a beneficial antiviral effect against SARS-CoV-2 although only at very large doses, suggesting that high exposures might be needed to achieve beneficial effects in treating COVID-19 [8, 9]. Therapeutic recommendations for the treatment of early COVID-19 still vary widely. Favipiravir has been recommended and was widely used as a treatment for COVID-19 in some countries, including Thailand (https://ddc.moph.go.th/viralpneumonia/eng/file/guidelines/g_treatment.pdf). Although some observational studies have suggested benefit from favipiravir [10–14], and a large clinical benefit was reported in one open-label randomised controlled trial (with shortening of time to clinical improvement from 14 to 2 days in hospitalised patients) [15], the other reported randomised trials have either shown no benefit, or the evidence of clinical efficacy has been marginal or unconvincing [16–29]. However, several of these studies were conducted in hospitalised patients, in whom the window of opportunity for antivirals to benefit may have closed. Antiviral drugs work better in early illness than in later infections in hospitalised patients where inflammatory pathology dominates. Dosing has also varied between the favipiravir studies. Given the lower antiviral activity of favipiravir against SARS-CoV-2 relative to influenza, high doses are probably necessary for optimal in vivo antiviral efficacy. Reassuringly no significant safety or tolerability issues have been identified in these clinical studies, although concerns have been raised regarding the risk to the fetus if potentially mutagenic antiviral nucleoside analogues are given to pregnant women [30].

Overall, the available evidence still leaves considerable uncertainty whether or not high-dose favipiravir is a useful antiviral treatment of early COVID-19 in outpatients. We present the results from a randomised platform trial assessing the in vivo antiviral activity of favipiravir in adults with acute early COVID-19.

## Methods

PLATCOV is an ongoing phase 2 open label, randomised, controlled adaptive platform trial (ClinicalTrials.gov: NCT05041907 registered 13/09/2021) [31]. It provides a standardised quantitative comparative method for in vivo assessment of potential antiviral treatments in low-risk adults with early symptomatic COVID-19. Daily oropharyngeal viral densities are measured by qPCR. The primary outcome measure in PLATCOV is the viral clearance rate derived from the slope of the log_10_ oropharyngeal viral clearance curve over the next 7 days following randomisation, estimated under a linear model [32]. The treatment effect is defined as the multiplicative change in viral clearance rate estimate relative to the contemporaneous no study drug arm (detailed below). The trial was conducted in Bangkok: Faculty of Tropical Medicine (FTM), Mahidol University, Bangplee hospital, Samut Prakarn; and Vajira hospital, Navamindradhiraj University, Bangkok, all in Thailand and in Belo Horizonte, Minas Gerais, Brazil (see Supplementary materials). All patients provided fully informed written consent. All methods were approved and carried out in accordance with local and national research boards in Thailand, the Mahidol University Faculty of Tropical Medicine Ethics Committee, the Central Research Ethics Committee, Thailand, the National Research Ethics Commission of Brazil, and the Oxford University Tropical Research Ethics Committee (see Supplementary materials). The PLATCOV trial was coordinated and monitored by the Mahidol Oxford Tropical Medicine Research Unit (MORU) in Bangkok, and overseen by a trial steering committee (TSC). Interim results were reviewed regularly by a data and safety monitoring board (DSMB). The funders had no role in the design, conduct, analysis or interpretation of the trial.

### Participants and procedures

Previously healthy adults aged between 18 and 50 years were eligible for the trial if they had early symptomatic COVID-19 (i.e., reported symptoms for ≤ 4 days), oxygen saturation ≥ 96%, were unimpeded in activities of daily living, and gave fully informed consent to study participation. SARS-CoV-2 positivity was defined either as a nasal lateral flow antigen test which became positive within two minutes (STANDARD® Q COVID-19 Ag Test, SD Biosensor, Suwon-si, Korea) or a positive PCR test within the previous 24h with a cycle threshold value (Ct) < 25 (all viral gene targets), both of which suggest high pharyngeal viral densities. The latter was added on 25 November 2021 to include those patients with recent PCRs confirming high viral loads. This was the only change to the pre-trial pre-specified inclusion/exclusion criteria. Exclusion criteria included taking any potential antivirals or pre-existing concomitant medications, chronic illness or significant comorbidity, haematological or biochemical abnormalities, pregnancy (a urinary pregnancy test was performed in females), breastfeeding, or contraindication or known hypersensitivity to any of the study drugs [31].

Block randomisation was performed for each site via a centralised web-app designed by MORU software engineers using RShiny®, hosted on a MORU webserver. At enrollment, after obtaining fully informed consent and entering the patient details, the app provided the randomised allocation. The no study drug arm comprised a minimum proportion of 20% of patients at all times, with uniform randomisation ratios applied across the active treatment arms. The study was open-label (no placebos). Enrolled patients were either admitted to the study ward (in Thailand), consistent with National recommendations at the time, or followed as outpatients at home (in Brazil). After randomisation and baseline procedures (see Supplementary materials) oropharyngeal swabs (two swabs from each tonsil) were taken as follows. Each flocked swab (Thermo Fisher MicroTest® and later COPAN FLOQSwabs®) was rotated against the tonsil through 360° four times and placed in Thermo Fisher M4RT™ viral transport medium (3mL). Swabs were transferred at 4–8°C, aliquoted, and then frozen at -80°C within 48h. Separate swabs from each tonsil were taken once daily from day 0 to day 7, and again on day 14. Each swab was processed and tested separately. Vital signs were recorded three times daily and symptoms and any adverse effects were recorded daily [31].

Patients allocated to favipiravir received 1800mg on an empty stomach, (nine 200mg tablets; Favir®, Government Pharmaceutical Organization in Thailand, *n* = 100; or Avigan®, FUJIFILM Toyama Chemical Co., Ltd. in Brazil *n* = 16), at the start of treatment followed 12 h later by a further 1800mg. Thereafter the patients took 800mg twice daily for a further 6 days totalling 13.2g over 7 days. All patients received standard symptomatic treatment excluding antivirals.

The TaqCheck® SARS-CoV-2 Fast PCR Assay (Applied Biosystems, Thermo Fisher Scientific, Waltham, Massachusetts) quantitated viral densities (SARS-CoV-2 RNA copies per mL). This multiplexed real-time PCR method detects the SARS-CoV-2 N and S genes, and human RNase P in a single reaction. RNase P was used to correct for variation in human cell content in samples. Viral densities were quantified against ATCC heat-inactivated SARS-CoV-2 (VR-1986HK strain 2019-nCoV/USA-WA1/2020) standards. Viral variants were identified using Whole Genome Sequencing (see Supplementary materials).

### Outcome measures

The primary outcome measure was the rate of viral clearance, expressed as a slope coefficient [32], and estimated under a Bayesian hierarchical linear model (mixed-effects model) fitted to the daily log_10_ oropharyngeal swab eluate viral density measurements between days 0 and 7 (18 measurements per patient). Before model fitting, Ct values were transformed to RNA copies per mL using a random effects linear model fit to the ATCC controls (random slope and intercept for each plate with additional fixed effects for each laboratory). Viral load measurements below the limit of quantification (Ct values ≥ 40) were treated as left-censored under the model. A non-linear model (allowing an initial log-linear increase in viral loads followed by a log-linear decrease in some patients) was also fitted to the data as a sensitivity analysis. All models included slope and intercept covariate effects for the virus variant, expressed as the major sub-lineages). Additional models included slope and intercept covariate effects for age, vaccination status, and days since symptom onset. The estimated individual viral clearance rates (i.e., slope coefficients from the model fit) can be expressed as clearance half-lives (t_1/2_ = log_10_ 0.5/slope). The treatment effect was defined as the multiplicative change (%) in the mean viral clearance rate relative to the no study drug arm (i.e., how much the test treatment accelerates on average the viral clearance) [32]. Thus, a 50% increase in clearance rate equals a 33% reduction in clearance half-life. All-cause hospitalisation for clinical deterioration (until day 28) was a secondary endpoint. For each studied intervention the sample size was adaptive based on prespecified futility and success stopping rules. Initially the futility stopping rule was set as a probability > 0.9 that the acceleration in viral clearance was < 5%, but at the prespecified open first interim analysis performed after 50 patients had been enrolled, the futility threshold was increased to 12.5%.

Adverse events were graded according to the Common Terminology Criteria for Adverse Events v.5.0 (CTCAE). Summaries were generated if the adverse event was ≥ grade 2 and was new or had increased in intensity. Serious adverse events were recorded separately and reported to the DSMB.

### Statistical analysis

All analyses were done in a prespecified modified intention-to-treat (mITT) population, comprising patients who had ≥ 3 days follow-up data. A series of linear and non-linear Bayesian hierarchical models were fitted to the viral quantitative PCR (qPCR) data (Supplementary materials). Model fits were compared using approximate leave-one-out comparison as implemented in the package *loo*. All data analysis was done in R version 4.0.2. Model fitting was done in S*tan* via the RS*tan* interface. All code and data are openly accessible via GitHub: https://github.com/jwatowatson/PLATCOV-Favipiravir.

## Results

The trial began recruitment on 30 September 2021. On 31 October 2022, the favipiravir arm of the trial was stopped and favipiravir was removed from the randomisation lists in Thailand and Brazil following a recommendation from the DSMB as the prespecified futility margin had been reached. This decision was based on PCR data from 102 patients randomised to favipiravir and 104 concurrent controls. Of the 615 patients enrolled by that time, 116 patients had been randomised to receive favipiravir, 132 had been randomised to no study drug, and the remainder (*n* = 367) were randomised to other interventions (casirivimab/imdevimab, tixagevimab/cilgavimab, remdesivir, ivermectin, nitazoxanide, fluoxetine, molnupiravir, or nirmatrelvir/ritonavir).

### Virological responses

**Fig. 1:**
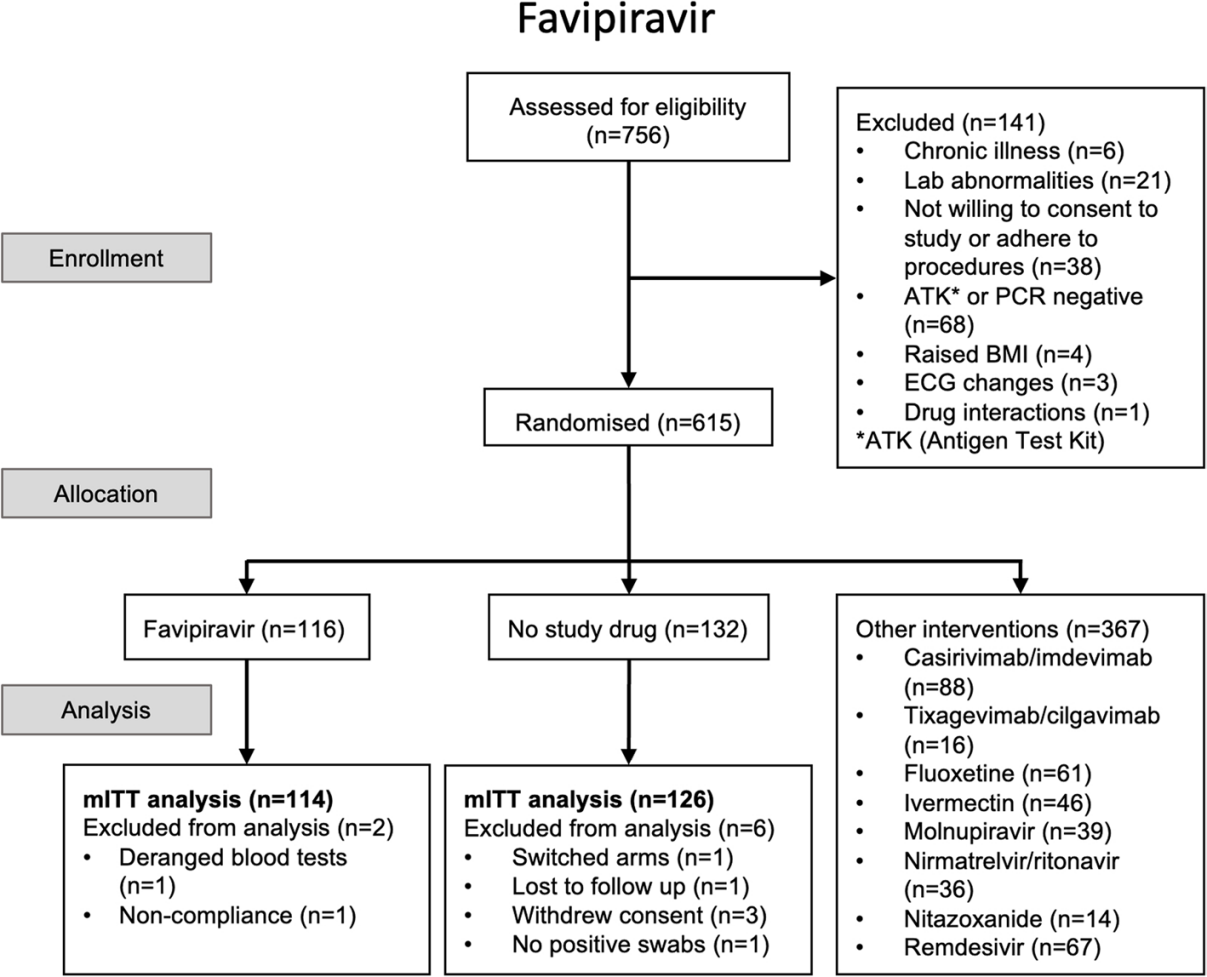
CONSORT diagram

**Fig. 2:**
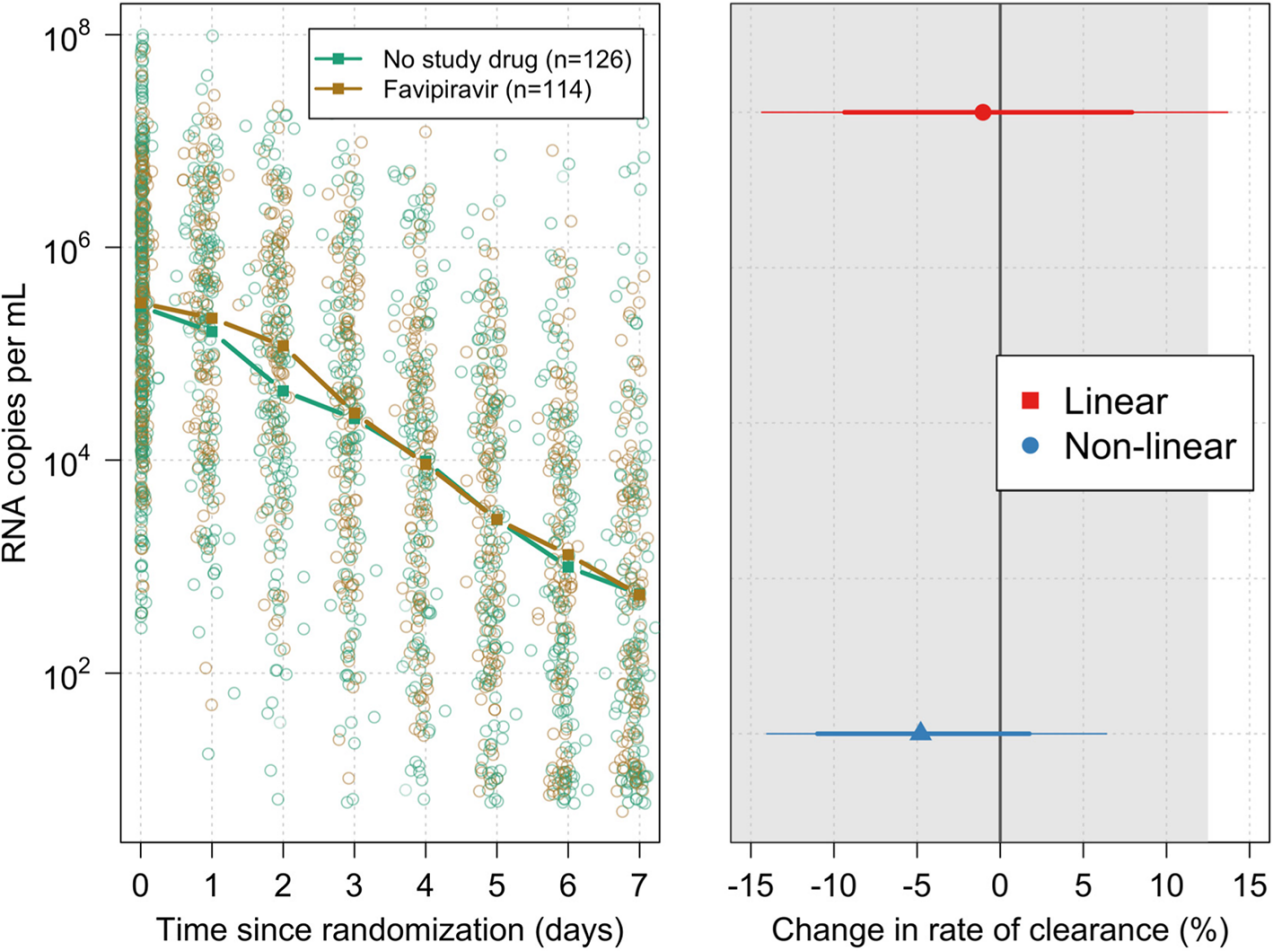
**a** (left): qPCR estimates of oropharyngeal swab eluate viral densities (all measurements) with the daily median values graphed by treatment arm (green: no study drug; brown: favipiravir). **b** (right): Estimated change in the rate of viral clearance under the linear (red) and non-linear (blue) models (median posterior estimates and corresponding 80% (thick line) and 95% (thin line) credible intervals are shown)

The mITT population included 114 patients randomised to favipiravir and 126 patients randomised to no study drug (Fig. 1). The baseline geometric mean (GM) oropharyngeal swab eluate viral load was 5.5×10^5^ RNA copies/mL (IQR 4.7×10^5^ to 6.3×10^5^), (Table 1, Fig. 2a). Rates of viral clearance were estimated under a linear mixed-effects model fit to all PCR data taken up to day 7 after randomisation in the mITT population (4,318 swabs in 240 patients, of which 3,839 were above the lower limit of quantification, 89%). A non-linear model was used as a sensitivity analysis. Under the linear model, there was no evidence of a difference in viral clearance rates between the favipiravir treated patients and those receiving no study drug (mean difference: –1%; 95%CI: -14% to 14%). The posterior probability that the effect was less than the pre-specified futility margin of 12.5% was 0.97 (Fig. 2b). The non-linear model gave very similar estimates (mean difference: -5%; 95%CI: -14% to 6%; probability less than 12.0% equal to 1).

**Table 1:** Baseline demographic characteristics in the mITT population

Baseline demographic characteristics in mITT population				
		No study drug	Favipiravir	Total
Total		126	114	240
Site n (%)	Brazil site	14 (11.1)	16 (14.0)	30 (12.5)
	Thailand FTM site	107 (84.9)	96 (84.2)	203 (84.6)
	Thailand Vajira site	3 (2.4)	2 (1.8)	5 (2.1)
	Thailand Bangplee site	2 (1.6)	0 (0.0)	2 (0.8)
Age (years)	Mean (SD)	30.0 (7.3)	30.2 (7.5)	30.1 (7.4)
Sex n (%)	Female	81 (64.3)	71 (62.3)	152 (63.3)
	Male	45 (35.7)	43 (37.7)	88 (36.7)
BMI (kg/m^2^)	Mean (SD)	23.0 (3.8)	23.1 (3.7)	23.0 (3.8)
Weight (kg)	Mean (SD)	62.8 (13.1)	63.0 (13.6)	62.9 (13.3)
Baseline viral load (log_10_ copies per mL)	Mean (SD)	5.4 (1.2)	5.5 (1.0)	5.5 (1.1)
SARS-CoV-2 Variant/ subvariants	Delta	10 (7.9)	11 (9.6)	21 (8.8)
	BA. 1	15 (11.9)	21 (18.4)	36 (15.0)
	BA. 2	58 (46.0)	47 (41.2)	105 (43.8)
	BA. 3	1 (0.8)	0 (0.0)	1 (0.4)
	BA. 4	2 (1.6)	3 (2.6)	5 (2.1)
	BA. 5	40 (31.7)	32 (28.1)	72 (30.0)
Symptoms duration (days)	Mean (SD)	2.2 (0.8)	2.1 (0.7)	2.2 (0.7)
Vaccinated (%)	Yes	122 (96.8)	112 (98.2)	234 (97.5)
	No	4 (3.2)	2 (1.8)	6 (2.5)

**Fig. 3:**
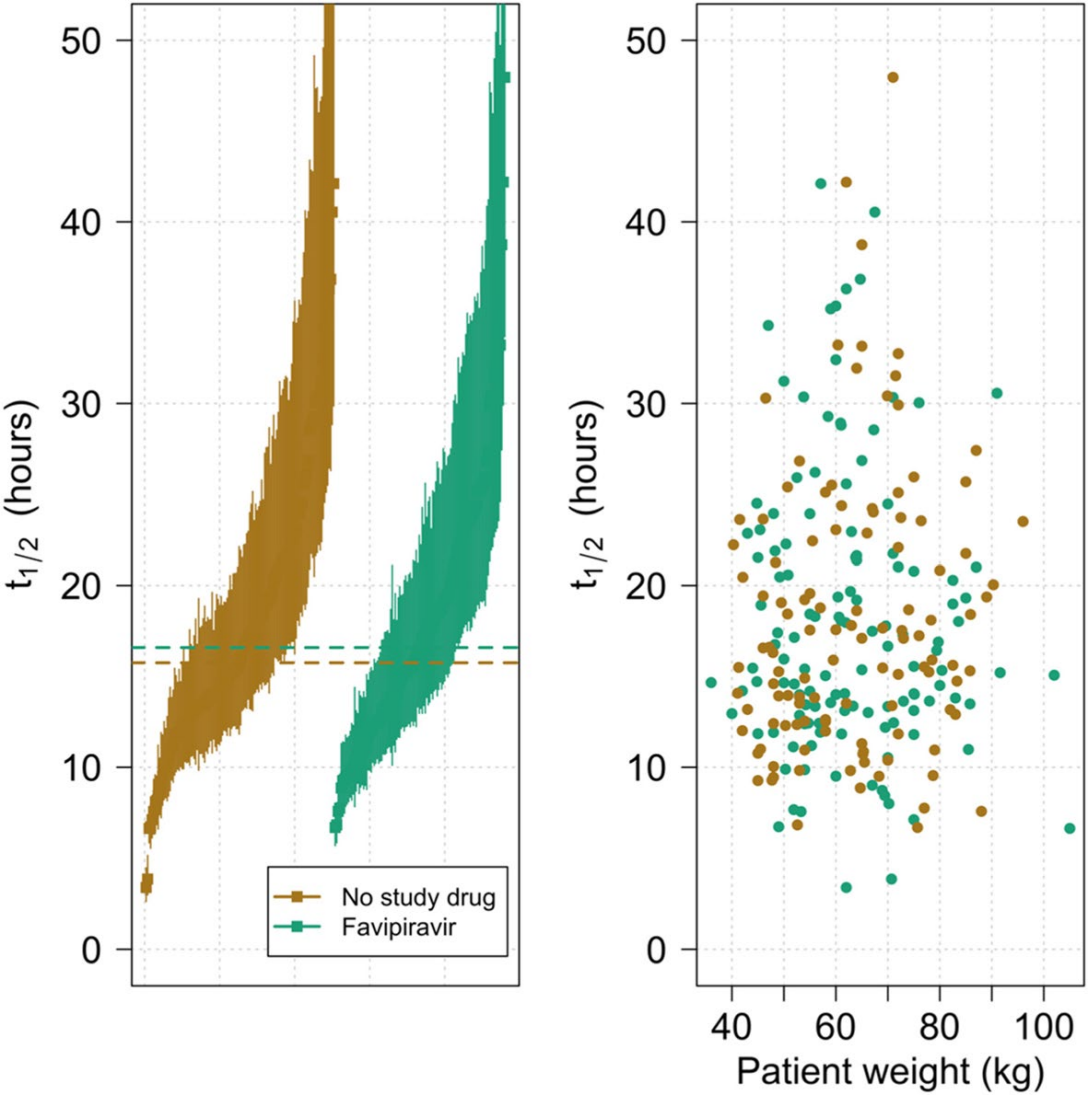
**a** (left): Estimated viral clearance half-lives ordered by increasing median estimate (lines show 80% credible intervals). **b** (right): Relationship between body weight and median estimated viral clearance half-life. As the individual doses were all the same, body weight is a surrogate for dose/kg and thus exposure

Under the linear model, patients treated with favipiravir had an estimated median viral clearance half-life of 16.6 h (range 6.7 to 48.0) and patients randomised to the no study drug arm had an estimated median viral clearance half-life of 15.7 h (range 3.4 to 42.1), (Fig. 3a). In patients receiving favipiravir, there was no association between body weight (i.e., mg/kg dose of favipiravir) and the estimated viral clearance (*p* = 0.2) (Fig. 3b).

### Adverse effects

The oropharyngeal swabbing procedures and all treatments were well-tolerated. There were three serious adverse events (SAEs) in the no study drug arm and two in the favipiravir arm, all resulting in the secondary endpoint of clinical deterioration leading to hospitalisation for medical reasons (three patients with raised creatinine phosphokinase (CPK) were already inpatients for isolation reasons; two no study drug and one favipiravir). In the favipiravir arm, a patient was readmitted 2 days after completing the 7-day course of favipiravir with fever and a maculopapular rash over the face, trunk, back, and extremities with sparing of the palms and soles. The rash was reviewed by a dermatologist who diagnosed a viral exanthem not related to the study drug. Two patients in the no study drug arm and one in the favipiravir arm had raised creatinine phosphokinase (CPK) levels (> 10 times ULN) attributed to COVID-19-related skeletal muscle damage. These improved with fluids and supportive management and were considered unrelated to study treatment. One patient in the no study drug arm was readmitted one day after discharge due to chest pain and lethargy. All clinical and laboratory investigations were normal and the patient was discharged the following day. There were no treatment related serious adverse events.

## Discussion

Continued uncertainty over the value of different COVID-19 treatments has resulted in substantial variation in therapeutic guidelines and clinical practices across the world. In the absence of other affordable and available oral antiviral treatments favipiravir has been recommended for the treatment of uncomplicated COVID-19 in several countries including Japan, Russia, Saudi Arabia, Turkey, Hungary, Kenya and Thailand (where it was recommended for patients with mild COVID-19 pneumonia from May 2020 until December 2022) (https://ddc.moph.go.th/viralpneumonia/eng/file/guidelines/g_treatment.pdf).

Knowing definitively if an antiviral drug has antiviral efficacy in vivo should be a prerequisite for its deployment. But the urgency and gravity of the spreading pandemic in 2020 meant that many drugs were recommended without clear evidence of clinical benefit. In this fourth year of the COVID-19 pandemic, increasingly mild clinical presentations resulting from immune protection from vaccines and previous infections, declining viral virulence, and availability in some regions of newly developed oral antivirals with proven efficacy (notably molnupiravir and nirmatrelvir/ritonavir) [33, 34], have all contributed to favipiravir being no-longer recommended for COVID-19. For the same reason use of other repurposed drugs has also decreased. This has left substantial uncertainty as to their clinical benefit in COVID-19, and their potential use in future pandemics caused by novel viruses.

This comparative in vivo pharmacodynamic assessment conducted in “low risk” adults with early symptomatic COVID-19 infections shows that favipiravir, given at relatively high oral doses, does not have measurable antiviral activity in vivo and is, therefore, very unlikely to be clinically beneficial. The lack of demonstrable in vivo activity contrasts with the approximate 30 to 40% acceleration in viral clearance rate observed for remdesivir and molnupiravir in this trial platform [31]. The main limitation of our study that it is open label, which may have led to more withdrawals in the no study drug arm.

Favipiravir was well-tolerated at the high doses used in this study. Favipiravir has complex non-linear pharmacokinetic properties [32]. It is metabolised primarily in the liver by aldehyde oxidase and excreted via the kidneys. Because of dose and time dependent auto-inhibition of aldehyde oxidase, favipiravir boosts its own plasma concentrations. This can result in exposures over twice the SARS-CoV-2 in vitro EC_90_ [6], although there is substantial inter-patient variability in achieved plasma concentrations, and lower exposures have been noted in certain populations, e.g. those from the United States compared to Japan and China [35]. Despite pharmacokinetic modelling suggesting that exposures sufficient for an antiviral effect can be achieved, the relationship between ex vivo SARS-CoV-2 inhibitory concentrations and consequent therapeutic effects in COVID-19 in vivo is uncertain. This study does not exclude therapeutic benefit from even higher oral or parenteral doses of favipiravir, although there was no evidence of a dose response relationship in this study derived from the variation in weight adjusted doses.

Similar negative results have been reported recently with ivermectin [36], which also fails to halt disease progression when given to outpatients [37]. In contrast, the antiviral remdesivir clearly does accelerate viral clearance [38], and in clinical trials it does prevent disease progression [39]. The association between accelerated viral clearance and improved clinical outcomes in early COVID-19 has been confirmed in studies with monoclonal antibodies as well as the newly developed antiviral drugs [33, 34, 36, 40–42]. In contrast, the reported lack of demonstrable antiviral effect in the PINETREE study of remdesivir, despite demonstration of a clear clinically beneficial effect, likely resulted from too infrequent nasopharyngeal viral density measurements and from the statistical analysis approach used to assess differences in viral loads. All these studies were completed in largely unvaccinated populations at a time when a higher proportion of COVID-19 infections progressed to hospitalisation and severe outcomes. If repeated today such studies would need to be substantially, and perhaps prohibitively, larger in order to detect clinical benefit. For example, molnupiravir was shown to provide clinical benefit in studies conducted over two years ago [33], but in the more recent community based PANORAMIC study [43] conducted in the UK there was no clear effect of molnupiravir on hospitalisation or death, despite recruiting 26,411 patients. However, molnupiravir was associated with a reduced time to recovery (although it was an open-label study) and faster reduction in viral loads. Given the very low event rate for the primary endpoint, despite its size, the PANORAMIC study was still underpowered.

The time and expense required to conduct large phase III studies in vaccinated populations and the difficulty of demonstrating efficacy using clinical end-points in early infections suggests that other approaches are needed for therapeutic assessment in COVID-19 (and other viral respiratory infections). The simple methodology described in this study provides one possible solution. It is readily performed anywhere which can perform accurate qPCR viral quantitation and it gives a rapid comparative assessment with much lower patient numbers than clinical trials with currently used viral endpoints (e.g. time-to-clearance) [44]. Duplicate daily oropharyngeal swabs are well-tolerated (whereas daily nasopharyngeal swabbing is not). The pharmacometric assessment can be used to characterise in vivo antiviral efficacy in real-time and thereby inform choice of drugs for large trials and therapeutic practice. Regulatory authority and treatment guideline decisions should be based upon evidence of in vivo antiviral efficacy, as well as in vitro evidence.

### Supplementary Information

**Additional file 1:** **Supplementary Table 1. **Summary of Adverse Events (grade 3 and above) for favipiravir. **Supplementary Table 2.** Summary of Serious Adverse Events. **Figure S1.** Genotyped SARS CoV2 variants over time (combined Thai and Brazilian sites). **Figure S2.** Covariate effects on intercept (left) and slope (right) for the linear model with additional covariate adjustment.

## Data Availability

All code and data are openly accessible via GitHub: https://github.com/jwatowatson/PLATCOV-Favipiravir. The final datasets will be stored locally and securely at the Mahidol Oxford Research Unit for long-term storage and access. Additional anonymised participant data can be made available by request on a case-by-case basis from the MORU Data Access Committee at datasharing@tropmedres.ac and can be made available by request to the corresponding author.
