# Supplementary material for: Clinical antiviral efficacy of favipiravir in early COVID-19 (PLATCOV): an open-label, randomised, controlled, adaptive platform trial
Source: BMC Infect Dis. 2024 Jan 15;24:89. doi: 10.1186/s12879-023-08835-3 (PMC10789040; doi:10.1186/s12879-023-08835-3)
Supplement: Supplementary file 1 — Additional file 1: Supplementary Table 1. Summary of Adverse Events (grade 3 and above) for favipiravir. Supplementary Table 2. Summary of Serious Adverse Events. Figure S1. Genotyped SARS CoV2 variants over time (combined Thai and Brazilian sites). Figure S2. Covariate effects on intercept (left) and slope (right) for the linear model with additional covariate adjustment. [file 12879_2023_8835_MOESM1_ESM.docx]

**Clinical antiviral efficacy of favipiravir in early COVID-19 (PLATCOV): an open-label, randomised, controlled adaptive platform trial**

[List of Sites and Investigators (PLATCOV Collaborative Group) 2](#_Toc127892287)

[Ethics Approval 5](#_Toc127892288)

[Baseline procedures 5](#_Toc127892289)

[Adverse events (AE) for favipiravir 6](#_Toc127892290)

[Serious Adverse Events 8](#_Toc127892291)

[Virus variant determination 9](#_Toc127892292)

[Randomisation 12](#_Toc127892293)

[Statistical Analysis 14](#_Toc127892294)

[Supplementary Results 16](#_Toc127892295)

[References: 16](#_Toc127892296)

# List of Sites and Investigators (PLATCOV Collaborative Group)

**Sites**

1. Hospital for Tropical Diseases (HTD), Faculty of Tropical Medicine, Mahidol University, 420/6 Rajvithi Road, Bangkok, 10400, Thailand
2. Vajira Hospital (VJ), Navamindradhiraj University, 681 Samsen road, Dusit, Bangkok, 10300, Thailand
3. Bangplee Hospital (BP), 88/1 Moo 8 Tambon Bang Phli Yai, Amphoe Bangplee, Samut Prakan 10540, Thailand
4. Universidade Federal de Minas Gerais, Belo Horizonte, Minas Gerais, Brazil

**Investigators**

Co-principal investigators:

Nicholas J White (nickw@tropmedres.ac)^1,2^

William HK Schilling (william@tropmedres.ac)^1,2^

Faculty of Tropical Medicine:

Site and Country Principal investigator:

Weerapong Phumratanaprapin^4^

Accountable Investigator:

Viravarn Luvira^4^

Co-Investigators:

James J Callery^1,2^

Nicholas PJ Day^1,2^

Sasithon Pukrittayakamee^1,4^

Simon Boyd^1,2^

Cintia Cruz^1,2^

Arjen M Dondorp^1,2^

Walter RJ Taylor^1,2^

James A Watson^1,2^

Watcharapong Piyaphanee^4^

Kittiyod Poovorawan^1,4^

Thundon Ngamprasertchai^4^

Tanaya Siripoon^4^

Borimas Hanboonkunupakarn^1,4^

Podjanee Jittamala^1,3^

Mallika Imwong^1,5^

Maneerat Ekkapongpisit^1^

Varaporn Kruabkontho^1^

Thatsanun Ngernseng^1^

Jaruwan Tubprasert^1^

Mohammad Yazid Abdad^1,2^

Srisuda Keayarsa^1^

Wanassanan Madmanee^1^

Runch Tuntipaiboontana^1^

Shivani Singh^1,2^

Kesinee Chotivanich^1,2^

Vajira Hospital:

Site Principal investigator:

Vasin Chotivanich^7^

Co-investigators:

Chunlanee Sangketchon^8^

Wiroj Ruksakul^7^

Bangplee Hospital:

Site Principal investigator:

Pongtorn Hanboonkunupakarn^6^

Co-investigator:

Sakol Sookprome^6^

Brazil Site:

Mauro M Teixeira^9^

Pedro J Almeida^9^

Renato S Aguiar^10^

Franciele M Santos^10^

**Affiliations:**

1. Mahidol Oxford Tropical Medicine Research Unit, Faculty of Tropical Medicine, Mahidol University, Bangkok, Thailand

2. Centre for Tropical Medicine and Global Health, Nuffield Department of Medicine, Oxford University, Oxford, UK

3. Department of Tropical Hygiene, Faculty of Tropical Medicine, Mahidol University, Bangkok, Thailand

4. Department of Clinical Tropical Medicine, Faculty of Tropical Medicine, Mahidol University, Bangkok, Thailand

5. Department of Molecular Tropical Medicine and Genetics, Faculty of Tropical Medicine, Mahidol University, Bangkok, Thailand

6. Bangplee Hospital, Ministry of Public Health, Thailand

7. Faculty of Medicine, Navamindradhiraj University, Bangkok,Thailand

8. Faculty of Science and Health Technology, Navamindradhiraj University, Bangkok,Thailand

9. Clinical Research Unit, Center for Advanced and Innovative Therapies, Universidade Federal de Minas Gerais, Brazil

10. Department of Genetics, Ecology and Evolution, Institute of Biological Sciences, Universidade Federal de Minas Gerais

# Ethics Approval

The trial was approved by local and national research ethics boards in Thailand (Faculty of Tropical Medicine Ethics Committee, Mahidol University, FTMEC Ref: TMEC 21-058) and the Central Research Ethics Committee (CREC, Bangkok, Thailand, CREC Ref: CREC048/64BP-MED34), in Brazil by the Research Ethics Committee of the Universidade Federal de Minas Gerais (COEP-UFMG, Minas Gerais, Brazil, COEP-UFMG) and National Research Ethics Commission- (CONEP, Brazil, COEP-UFMG and CONEP Ref: CAAE:51593421.1.0000.5149), and by the Oxford University Tropical Research Ethics Committee (OxTREC, Oxford, UK, OxTREC Ref: 24-21).

# Baseline procedures

Baseline investigations included a full clinical examination, rapid SARS-CoV-2 antibody test (BIOSYNEX COVID-19 BSS IgM/IgG®, Illkirch-Graffenstaden, France, done in Thailand only), blood sampling for hematology and biochemistry, an electrocardiogram and a chest radiograph (following local guidance in Thailand, but not a study requirement).

# Adverse events (AE) for favipiravir

**Supplementary table 1: Summary of adverse events (grade 3 and above) for favipiravir.**

|  | **All grades** | | **Grade 3-4** | |
| --- | --- | --- | --- | --- |
|  | **Favipiravir**  **(n=116)** | **No study drug (n=132)** | **Favipiravir**  **(n=116)** | **No study drug (n=132)** |
| Any adverse event (Grade ≥ 3) | 2 | 3 |  |  |
| Serious adverse event reported | 2 | 3 |  |  |
| Symptoms |  |  |  |  |
| Fever |  |  | 0 | 0 |
| Headache |  |  | 0 | 0 |
| Dizziness |  |  | 0 | 0 |
| Blurred vision |  |  | 0 | 0 |
| Fatigue |  |  | 0 | 1* |
| Cough |  |  | 0 | 0 |
| Difficulty breathing |  |  | 0 | 0 |
| Chest pain |  |  | 0 | 0 |
| Running nose |  |  | 0 | 0 |
| Loss of smell or taste |  |  | 0 | 0 |
| Abdominal pain |  |  | 0 | 0 |
| Loss of appetite |  |  | 0 | 0 |
| Nausea |  |  | 0 | 0 |
| Vomiting |  |  | 0 | 0 |
| Diarrhoea |  |  | 0 | 0 |
| Arthralgia |  |  | 0 | 0 |
| Myalgia |  |  | 0 | 0 |
| Itching |  |  | 0 | 0 |
| Skin rash |  |  | 1* | 0 |
| Laboratory abnormalites |  |  |  |  |
| Creatinine |  |  | 0 | 0 |
| BUN |  |  | 0 | 0 |
| Sodium |  |  | 0 | 0 |
| eGFR |  |  | 0 | 0 |
| Potassium |  |  | 0 | 0 |
| ALT/SGPT |  |  | 0 | 0 |
| AST/SGOT |  |  | 0 | 0 |
| Total bilirubin |  |  | 0 | 0 |
| Direct bilirubin |  |  | 0 | 0 |
| Alkaline Phosphatase |  |  | 0 | 0 |
| LDH |  |  | 0 | 0 |
| Creatinine phosphokinase (CPK) |  |  | 1* | 2* |
| Anemia |  |  | 0 | 0 |
| Leukocytopenia |  |  | 0 | 0 |
| Neutropenia |  |  | 0 | 0 |
| Thrombocytopenia |  |  | 0 | 0 |

**Patients were also classified as serious adverse events and are detailed in the serious adverse events table (Supplementary Table: 2).*

# Serious Adverse Events

**Supplementary table 2: Summary of Serious Adverse Events**

| **Number** | **Study arm** | **Final diagnosis** | **Relationship to trial drug** | **Resolved** |
| --- | --- | --- | --- | --- |
| 1 | No study drug | Reduction in activities of daily living after COVID-19 infection ^1^ | Not related | Yes |
| 2 | No study drug | COVID-19-related skeletal muscle damage ^2^ | Not related | Yes |
| 3 | No study drug | COVID-19-related skeletal muscle damage ^2^ | Not related | Yes |
| 4 | Favipiravir | COVID-19-related skeletal muscle damage ^2^ | Not related | Yes |
| 5 | Favipiravir | Viral exanthems^3^ | Not related | Yes |

*^1^Participant was impeded in activities of daily living one day post-discharge from the ward (day 8) and was readmitted for further investigation complaining of right-sided chest pain and lethargy. Clinical observations incl. oxygen saturations, physical examination and electrocardiogram were unremarkable, laboratory investigations including inflammatory markers and D-Dimers were in the normal range. A SARS-CoV-2 PCR was negative. CT pulmonary angiogram showed no radiological evidence of pulmonary embolus,or pneumonitis. The patient’s symptoms quickly resolved and the patient was discharged the following day.*

*^2^Participants had an acute rise in creatinine phosphokinase (CPK) during admission. Myoglobin was not detected on urinalysis. A diagnosis of COVID-induced rhabdomyolysis was made by the clinical team based on symptoms of myalgia and a raised CPK without another identifiable aetiology. Nephrology review advised supportive treatment only. Symptoms resolved, the CPK normalised and was within normal limits on follow-up.*

*^3^Participant was readmitted with high grade fever and 2 day history of rash starting after discharge. The rash was generalised maculopapular on the face, trunk, back and extremities (sparing palms and soles). There was bilateral posterior auricular and posterior cervical lymphadenopathies; and hepatomegaly without arthritis. She was negative for Dengue, Leptospirosis, CMV, HCV, HBV, measles, malaria, HIV, UTI, EBV, bacteraemia and rubella. Her AST was mildly raised on admission at 59, with an ALT of 29. WCC was 7.8, lymphocytes 10.0, neutrophils 7.2, eosinophils 0.0, platelets were 167. Treated with empirical ceftriaxone and doxycycline, anti-histamines and paracetamol. Reviewed by the dermatologist who did not feel the nature of the rash nor timing were in keeping with a drug reaction, advised supportive treatment.*

# Virus variant determination

Brazil site – Virus variant determination

SARS-CoV-2 whole-genome sequencing

The sequencing was carried out using two different technologies, Illumina (Illumina, USA) and IonTorrent (ThermoFisher Scientific, USA). Only SARS-CoV-2-positive samples with Ct < 30 values for virus targets were considered. Illumina libraries were prepared using the QIAseq FX DNA Library Prep kit (QIAGEN, Germany) and sequenced on the Illumina MiSeq platform (Illumina, USA) with a v3 (600 cycles) cartridge, following all manufacturer’s protocols. IonTorrent libraries were prepared using the Ion AmpliSeq SARS-CoV-2 Panel (ThermoFisher Scientific, USA) and sequenced on the Ion Torrent PGM platform with a 314-chip kit (ThermoFisher Scientific, USA), according to the manufacturer’s recommendations. Three negative controls were used in all sample processing steps (cDNA synthesis, viral genome amplification, and library preparation).

Viral genome assembly and classifications

A custom pipeline was used to process the sequencing data. In the first step, quality control was performed with Trimmomatic v0.39. Adapter and primer sequences, short reads (< 50 nucleotides), and low-quality bases (Phred score < 30) were removed. Next, reads were mapped against the SARS-CoV-2 reference genome (GenBank accession: NC_045512) with Bowtie2. Samtools manipulated the mapping files, whilst consensus genome sequences were estimated using the bcftools consensus option. Masking of low-coverage sites was performed with bedtools. The code for the described pipeline can be found on GitHub (https://github.com/filiperomero2/ViralUnity). Depth thresholds differed between sequencing technologies employed. For IonTorrent data, sites with less than 20-fold depth were masked, while for Illumina, the minimum threshold was 10-fold. Sequences <70% genome coverage breadth were removed from downstream analysis. Consensus sequences were classified using the NextClade web application v.1.7.0, or Pangolin tool v.3.1.11.

Thailand site – Virus variant determination

SARS-CoV-2 whole-genome sequencing

The sequencing method carried out in this experiment follows the “PCR tiling of SARS-CoV-2 virus with rapid barcoding and Midnight RT PCR Expansion” provided by Oxford Nanopore Technology (Oxford, UK) developed based on a protocol by ARTIC network group^1^. Library preparation process started with reverse transcription, which consists of mixing the purified viral RNA with LunaScript RT SuperMix and incubating the mixtures in a thermal cycler. DNA fragments to be used in the assembly process were amplified by PCR using Midnight primer set (V3) and attached with barcodes from Rapid Barcode Plate (RB96). The mixtures from each sample were pooled together, cleaned with AMPure XP Beads (AXP) and attached with Rapid Adapter F (RAP F). The prepared DNA fragments were then loaded into a primed flow cell (FLO-MIN106) and sequenced on GridION MK1 system.

Viral genome assembly and classification

The output sequencing data (.fast5) from MinKNOW software was base-called with Guppy software using the High Accuracy (HAC) model to generate nucleotide sequence data for each fragment (reads) in the fastq format. These base-called data were then processed through the established workflow wf-artic on EPI2ME software to be assembled into consensus sequences. Only reads with average Phred Quality (Q) score above 9 and minimum and maximum length of 250 and 1500 bps were used in the assembly process. The consensus sequences were then classified using the Pangolin tool (4.1.1) and Pangolin dataset (v1.14).

The virus variants over time are shown in Figure S1.


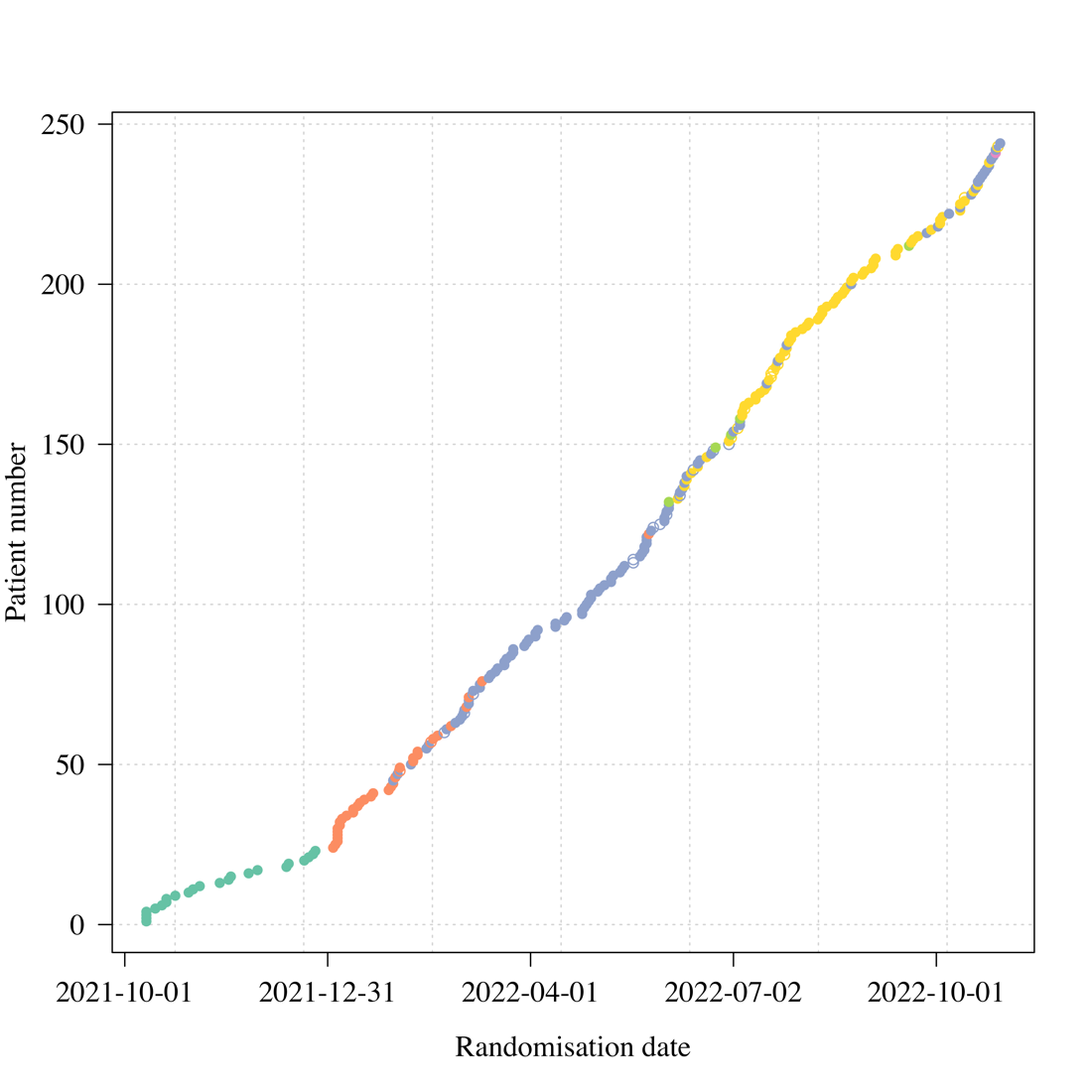


Figure S1 Genotyped variants over time (combined Thai and Brazilian sites). The data from Brazil are imputed based on date.

# Randomisation

The randomisation sheets were generated by the trial statistician (James Watson).

All new randomisation sheets and all updates of existing randomisation sheets were done using a pre-written R script which was stored on the randomisation Dropbox folder (owner is MORU, under custodianship of the head of MORU IT; this file is a full ‘Professional’ version with history recorded and only the trial statistician and head of IT had access. The file took the following inputs:

- Site codes (e.g. “th001”) for which to generate randomisation sheets;
- The set of arms available for randomisation in that site;
- The number of arm repeats per block (this is set to the minimum integer such that in each block there is an integer number for each arm);
- The randomisation data file from each site (which has the patient numbers for subjects already randomised) named data-XXX.csv (where XXX is the site code), if this does not yet exist a blank csv (headers only) is generated.

This R script is run every time a new site becomes active and every time the set of available arms changes. The output is a csv file named rand-XXX.csv (where XXX is the site code). This overwrites the pre-existing file (which can be retrieved from the Dropbox version history). Each time the randomisation script is run, this is recorded on a log file*.*

The randomisation is done according to the following constraints:

- Blocks of 2*number of available arms;
- Additional ‘fuzziness’ by swapping one patient allocation per block at random (this can be swapped for any of the available arms) – this avoids knowing which arm the last patient per block will receive.

Each time an authorised member of the study team logs onto the web-app this is logged (timestamp and username).

Each time a new patient is randomised this is logged on to the file data-XXX.csv (where XXX is the site code) with the following information:

- Subject number
- Screening number
- Age
- Sex
- Member of study team username
- Timestamp

# Statistical Analysis

The primary analysis consists of fitting Bayesian hierarchical (mixed effects) linear models to the serial log_10_ viral load data up until day 7 (the day 14 data were not used). All models encode residual error as a *t*-distribution with degrees of freedom estimated from the data. The *t*-distribution was chosen for robustness as the residual error is clearly non-Gaussian. The *t*-distribution error model also makes the inferences robust against model mis-specification (particularly for the linear models).^2^ All models include correlated individual random effect terms for both the intercept (baseline viral load) and the slope. All changes to the slope are defined as multiplicative changes on the log scale (i.e. a value of 0 equals no change).

The treatment effect is defined as the proportional change (expressed as a multiplicative term) in the population slope of the daily change in log_10_ viral load. The data are modelled on the log_10_ copies per mL scale, after conversion from Ct values using the standard curve generated from the 12 control concentrations (synthetic samples with known viral densities) from each 96 well plate. The standard curve transformation is done by fitting a linear mixed effects model (random slope and random intercept for each plate) to the control data: regressing the Ct values on the known log viral densities. This borrows information across plates and adjusts for batch effects.

For all models, we adjusted the intercept and slope for the enrolling site (4 sites in total, the reference site is the Hospital of Tropical Diseases which recruited >80% of patients) and for the variant called (Delta is reference: BA.1, BA.2, BA.4, BA.5 are the alternatives). A subset of models also adjusted the slopes and intercepts for:

- Age
- Number of vaccine doses
- Days since symptom onset

All models adjust for human RNase P (proxy for the number of human cells in the sample). This is an independent linear predictor for each viral load measurement. We fit the models using two sets of prior distributions: weakly informative priors (WIP) and non-informative priors (NIP).

***Models fitted***

For each analysis we fit 5 separate models:

1. **Model 1 is linear with RNase P adjustment; adjustment for site & variant; WIP. This is the main model used to report treatment effects.**
2. Model 2 is non-linear; RNase P adjustment; adjustment for site & variant; WIP
3. Model 3 is linear with RNase P adjustment; adjustment for site & variant; non-informative priors (NIP)
4. Model 4 is non-linear with RNase P adjustment; adjustment for site & variant; NIP
5. Model 5 is linear with RNase P adjustment; full covariate adjustment; WIP

Model 1 was used for all stopping decisions. All models have RNase P adjustment and are all combinations of linear & non-linear models, with or without full covariate adjustment; and with either weakly informative priors or non-informative priors. We compared model fits using the *loo* (approximate leave-one-out cross validation) package. The statistical analysis plan provides a detailed overview of the model structures.

1. ***Code and analysis plan***

All data, models and analytical output are on the linked GitHub repository: https://github.com/jwatowatson/PLATCOV-Favipiravir

The GitHub repository includes all data used in the analysis for full reproducibility of the results. The main Markdown file (Favipiravir_analysis.qmd) goes through all data manipulation and model fitting.

# Supplementary Results


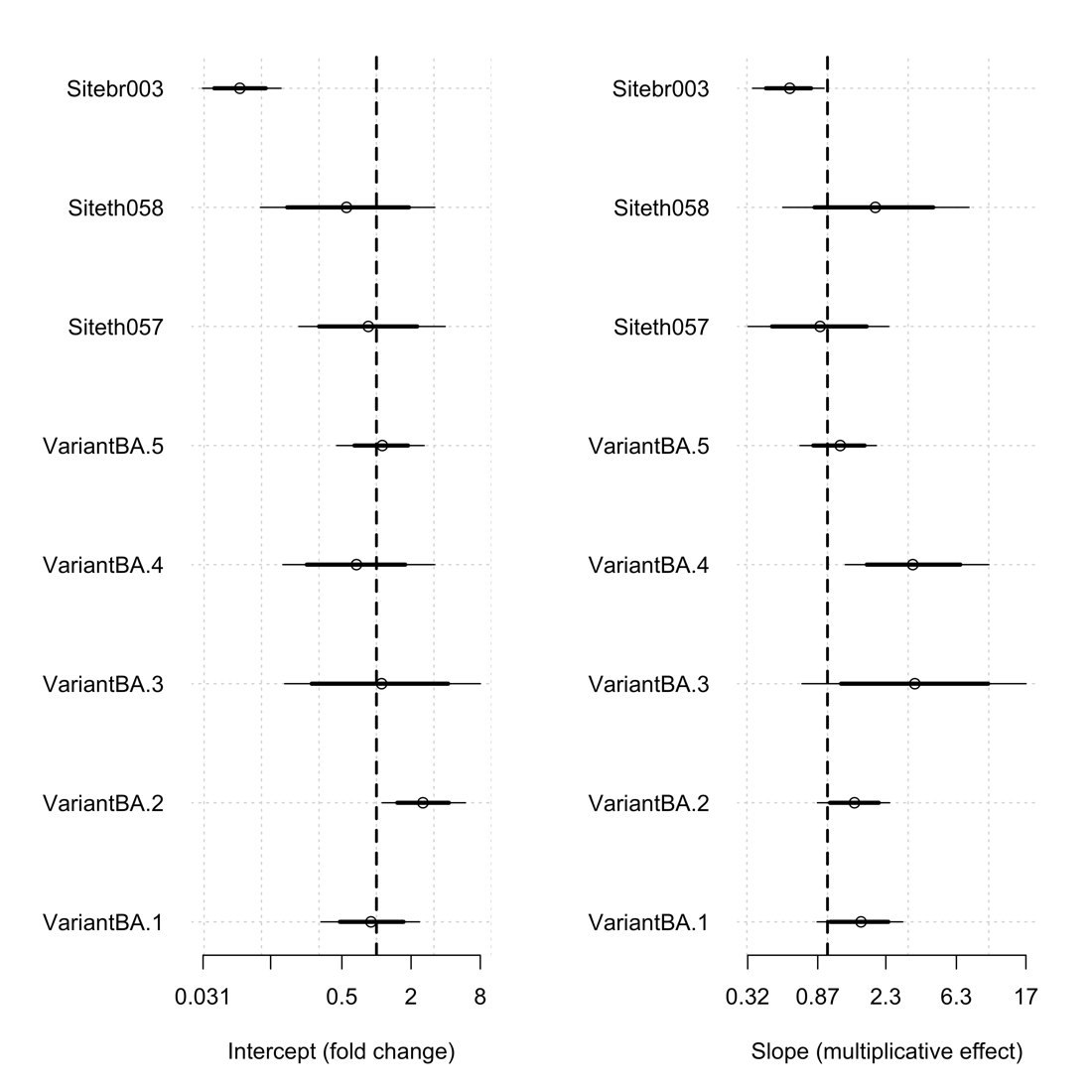


Figure S2 Covariate effects on intercept (left) and slope (right) for the linear model with additional covariate adjustment. Sites compared to HTD (Hospital of Tropical Diseases, Faculty of Tropical Medicine, Bangkok, Thailand), br003 (Brazil site), th058 (Bangplee Hospital), th057 (Vajira Hospital). Variants/subvariants compared with Delta variant.

# References:

1. Quick J. nCoV-2019 sequencing protocol v3 (LoCost) V.3. (2020). Available online at: https://www.protocols.io/view/ncov-2019-sequencing-protocol-v3-locost-bh42j8ye

2. Lange KL, Little RJA, Taylor JMG. Robust Statistical Modeling Using the t Distribution. J Am Stat Assoc. 1989. 84(408), 881–896. https://doi.org/10.2307/2290063
